# Musculoskeletal Complications in COVID-19: Exploring the Role of Key Biomarkers

**DOI:** 10.3390/ijms26178569

**Published:** 2025-09-03

**Authors:** Sagar Patel, Cameron Foster, Kamal Patel, Monte Hunter, Carlos M. Isales, Sadanand Fulzele

**Affiliations:** 1 Department of Medicine, Augusta University, Augusta, GA 30912, USA; spatel47@augusta.edu (S.P.); cisales@augusta.edu (C.M.I.); 2 Department of Orthopaedic Surgery, College of Medicine, University of Florida, Jacksonville, FL 32209, USA; 3 School of Dentistry, University of Alabama at Birmingham, 1919 7th Ave S, Birmingham, AL 35233, USA; kgpatel@uab.edu; 4 Department of Orthopaedic Surgery, Augusta University, Augusta, GA 30912, USA; mohunter@augusta.edu; 5 Center for Healthy Aging, Augusta University, Augusta, GA 30912, USA

**Keywords:** bone, musculoskeletal, COVID-19, biomarkers

## Abstract

The COVID-19 pandemic has revealed significant secondary complications affecting musculoskeletal (MSK) health, especially in patients with pre-existing conditions. This review synthesizes data from clinical and experimental studies on key MSK biomarkers, including cartilage oligomeric matrix protein (COMP), hyaluronic acid (HA), osteocalcin, alkaline phosphatase (ALP), procollagen type I N-terminal peptide (PINP), osteopontin (OPN), matrix metalloproteinases (MMP-3 and MMP-9), myostatin, IGF-1, follistatin, and creatine kinase. COVID-19 is associated with decreased COMP and osteocalcin levels, indicating cartilage degradation and impaired bone formation, alongside elevated HA, ALP, PINP, OPN, and MMPs, reflecting increased joint inflammation, bone remodeling, and tissue breakdown. Changes in myostatin, IGF-1, follistatin, and creatine kinase levels have been shown to be linked with COVID-19-related sarcopenia. These biomarker alterations provide insight into the underlying mechanisms of MSK damage in COVID-19 patients and highlight the potential for using these markers in early diagnosis and management of post-COVID musculoskeletal disorders. Further longitudinal research is essential to develop targeted therapies aimed at mitigating long-term MSK complications in affected individuals.

## 1. Introduction

The emergence of the novel coronavirus, SARS-CoV-2, in late 2019 marked the beginning of a global health crisis that would come to be known as COVID-19 [1]. This viral respiratory illness, characterized by a wide spectrum of symptoms ranging from mild respiratory discomfort to severe pneumonia and death, quickly spread across the globe, impacting millions of lives [2]. While the primary focus of research and clinical attention has understandably been on the respiratory manifestations of COVID-19, it has become increasingly clear that this disease can have far-reaching effects, including secondary complications, some of which are long-lasting, such as musculoskeletal (MSK) issues.

Susceptibility to COVID-19 is not uniform across populations, with the elderly and individuals with pre-existing chronic diseases being particularly vulnerable [3]. This susceptibility extends to MSK pathology, as the aging population is highly susceptible to both COVID-19 and various MSK problems [4,5,6]. As the virus continued to spread rapidly, studies were primarily directed towards understanding its impact on the respiratory system and finding effective treatments and vaccines [7,8,9,10]. However, it became evident that there was a need to explore the secondary complications of long COVID-19, especially those affecting the musculoskeletal system, which can have serious and enduring consequences for individuals exposed to the virus over an extended period [11,12,13]. While substantial studies have been conducted on COVID-19, there is a notable gap in the literature regarding prospective studies examining MSK biomarkers and their correlation with COVID-19. In this study, we aim to address this gap by summarizing the MSK-related signaling pathway (biomarkers) that are dysregulated in COVID-19 patients and exploring their potential connection to MSK pathophysiology.

This study will primarily focus on biomarkers, drawing upon data from studies involving patients with serum-based assessments. While the most common biomarkers for MSK issues are non-specific inflammatory markers, it is important to note that other biomarkers can also indicate different MSK problems. We will delve into a comprehensive analysis of serum biomarkers in COVID-19 patients and explore their potential implications for musculoskeletal health. By doing so, we aim to shed light on an area of COVID-19 research that has yet to be extensively explored, thereby contributing to a more comprehensive understanding of the long-term effects of this pandemic on human health.

## 2. Method

This manuscript is a narrative review that synthesizes current evidence on musculoskeletal biomarkers associated with COVID-19. Relevant literature was identified through a non-systematic search of PubMed, Scopus, and Web of Science up to [December 2019–June 2025], using combinations of keywords such as “COVID-19,” “SARS-CoV-2,” “musculoskeletal,” “biomarkers,” “bone,” “cartilage,” “muscle,” and the specific biomarker names (e.g., “COMP,” “hyaluronic acid,” “osteocalcin,” “myostatin”). Additional articles were identified through manual screening of reference lists from key publications. Both clinical and experimental studies were considered. Priority was given to peer-reviewed, English-language articles, with emphasis on studies that directly measured musculoskeletal biomarkers in COVID-19 contexts. No formal inclusion/exclusion criteria or systematic review protocols (e.g., PRISMA) were applied, as the purpose of this review is to provide an integrative synthesis of the available literature rather than a quantitative meta-analysis. The evidence gathered was critically evaluated and organized by biomarker category to highlight observed trends, underlying mechanisms, and potential clinical implications.

## 3. COVID-19 and Musculoskeletal Pathology

The complications associated with COVID-19 included many secondary systems, including a high prevalence of fatigue and muscle pain [14]. Recent clinical evidence has demonstrated that COVID-19 has significant and multifaceted impacts on the musculoskeletal system, particularly in relation to bone and cartilage health. In a study by a Turkish group, quantitative CT (QCT) was used to find an average decrease in BMD of 8.6% between diagnoses of COVID-19 and follow-up scans. This decrease in BMD caused the number of patients who were considered osteoporotic to double after being hospitalized due to COVID [15]. For context, the average age-related annual BMD loss is approximately 0.75% in women and 0.86% in men [16], highlighting the disproportionate bone loss associated with COVID-19. Additional studies have reported that lower BMD in COVID-19 patients is associated with increased disease severity and higher mortality risk and that vertebral fractures are highly prevalent among hospitalized COVID-19 patients, further linking COVID-19 to increased fracture risk and bone fragility [17]. Krenytska et al. showed that patients with osteoarthritis (OA) who had recovered from COVID-19 exhibited elevated plasma levels of IL-1β and a reduction in angiogenesis-related growth factors such as VEGF and PDGF, suggesting a persistent pro-inflammatory state and impaired tissue repair that may accelerate cartilage loss and OA progression. Additionally, these patients had a significant decrease in the stress protein HSP60, which is associated with cellular homeostasis and cartilage protection, further implicating COVID-19 in joint degeneration [18].

Muscle loss and acute sarcopenia are also well-documented sequelae of COVID-19. Gobbi et al. reported that 58% of post-acute COVID-19 patients admitted for rehabilitation met the criteria for sarcopenia, with significant reductions in muscle mass, handgrip strength, and functional capacity at admission [19]. Piotrowicz et al. described how the hyperinflammatory state, physical inactivity, and nutritional deficits associated with COVID-19 contribute to rapid muscle wasting, with patients at risk of losing 5–10% of their body weight during the acute phase, primarily due to muscle loss [20]. Piotrowicz et al. further observed that post-COVID-19 patients without prior disabilities had biceps and quadriceps strength at only 69% and 54% of predicted values, respectively [20]. Collectively, these findings emphasize that post-COVID-19 patients are at increased risk for osteoarthritis progression, cartilage and bone loss, and significant muscle wasting, all of which may contribute to long-term functional impairment and increased fracture risk.

Similar to the decreased BMD observed in patients, animal models have provided supporting evidence for COVID-19–associated skeletal pathology. In a study by Bobin Mi and group, it was found that mouse models showed osteoporotic characteristics of post-COVID infection. The animal model also identified delayed fracture healing [21]. The COVID-19 virus is negatively affecting osteogenic differentiation, leading to the decreased BMD seen in many hospitalized COVID-19 patients. This study aims to investigate the correlation between COVID-19 and alterations in MSK-related biomarkers in order to gain mechanistic insights into the impact of SARS-CoV-2 on musculoskeletal health. By analyzing changes in specific molecular and biochemical markers associated with muscle and bone physiology, the study seeks to identify predictive biomarkers and delineate potential pathways through which COVID-19 may contribute to MSK dysfunction, ultimately enabling the prediction of MSK pathology in affected individuals.

## 4. Alteration in Cartilage Biomarkers Post-COVID-19

Post-COVID-19 joint pain, particularly affecting the knee, shoulder, and hip, has been reported in patients following infection [22]. Acute joint pain is a known clinical feature in several viral infections, including COVID-19 [23,24,25]. Chronic joint pain is rare after the viral infection, but in COVID-19, the percentage of patients experiencing it is higher [23]. Serum metabolomics studies on individuals with long COVID have demonstrated alterations in cartilage degradation biomarkers, resembling those seen in osteoarthritis patients. The two-cartilage biomarkers cartilage oligomeric matrix protein (COMP) and Hyaluronic Acid (HA) are important biomarkers for understanding the pathological changes occurring in the cartilage.

### 4.1. Cartilage Oligomeric Matrix Protein (COMP)

The cartilage oligomeric matrix protein (COMP) is a non-collagenous protein of articular cartilage [26]. It is a pentameric non-collagenous glycoprotein and a member of the family of thrombospondin, which acts as a catalyst in collagen formation [27]. COMP functions to bind to type II collagen fibers and stabilize the collagen fiber network in the articular cartilage [28]. It has been shown to be a prominent constituent of articular cartilage. Increased serum concentrations of COMP fragments have been reported for patients with knee osteoarthritis (OA) and early rheumatoid arthritis (RA). It has thus been suggested that patients with high levels of COMP in serum might represent individuals with increased articular cartilage degradation. Indeed, patients with greater serum COMP concentration experience a faster progression of their disease [29]. Patients with knee OA have been shown to have elevated levels of COMP, which has been sensitive to the severity of the disease [30]. According to Rousseau, the BIPED classification associates COMP levels with diagnosis, disease burden, and prognosis of OA [26]. In a cohort study of women who were followed for 20 years, serum COMP levels were predictive of the occurrence of painful OA and structural changes to their knees [31]. A case–control study of 150 patients performed by Verma showed the use of COMP in assessing the risk of rapid progression of knee OA in patients [32]. Serum COMP has proven to be a valuable indicator that can be used as a diagnostic marker.

Individuals who have been diagnosed with COVID-19 have been shown to have decreased serum COMP levels. In a recent study, Huet et al. investigated the expression of the cartilage oligomeric matrix protein (COMP) gene in the blood of patients with osteoarthritis following recovery from SARS-CoV-2 infection. Their findings revealed a significantly greater increase in COMP levels in post-COVID-19 osteoarthritis patients compared to those with primary knee osteoarthritis, suggesting a potential exacerbating effect of SARS-CoV-2 on cartilage metabolism and joint pathology [33]. In a retrospective cohort study, there were a total of 455 participants which were divided into groups based on the severity of the disease. In this study, patients with more severe disease were shown to have downregulated COMP levels in serum [34]. This may be due to the virus itself or many other factors, including medication. A recent treatment approved by the FDA for emergency use with COVID-19 patients is Paxlovid. It is an antiviral drug combination of nirmatrelvir and ritonavir. The work completed by Kong in mouse models indicated how Paxlovid accelerates the degradation of cartilage and OA development by inhibiting chondrogenic differentiation [35]. This indicates the importance of follow-up visits with patients and identifying levels of COMP to ensure that the severity of OA does not increase.

### 4.2. Hyaluronic Acid (HA)

Hyaluronic Acid (HA) is a non-sulfated glycosaminoglycan that is abundantly found in articular cartilage, synovial fluid, and endothelial matrix. It plays a role in the lubrication and viscoelasticity of the synovial while regulating several processes within the joint space and structures [27]. The synovial fluid is present in joint capsules to reduce friction between the articular cartilages. For this reason, synovial fluid must maintain its viscosity to be an effective lubricant. HA helps maintain the viscosity of the fluid to allow articulating joints to move smoothly. However, in certain states, when there is increased inflammation or oxidative stress, HA begins to be degraded, causing several joint-related diseases. The articular cartilage in these diseases is greatly affected because HA plays a role in nutrients getting to the cartilage, along with keeping the joint cavity open to allow extended movements. Due to the importance of HA, it is continuously secreted and removed by the synovium [36]. According to Rousseau, of the biomarkers they evaluated, HA was associated with the progression of knee and hip OA. HA was elevated in both of these disease states [26]. In patients with knee OA, the HA chain length and concentration were reduced, indicating low viscosity of the synovial fluid, which propagates the damage of cartilage associated with the disease [36]. HA can be an effective biomarker because levels of it can indicate severity of disease as well as asymptomatic versus symptomatic cases of OA [37,38].

COVID-19 has been associated with significant alterations in hyaluronan (HA) levels, possibly contributing to the development of arthritic issues in affected patients. The infection leads to a dysregulation of HA metabolism, characterized by accumulating HA fragments in the lungs and systemic circulation, particularly in severe cases [39,40]. This accumulation is linked to endothelial dysfunction and increased vascular permeability, exacerbating inflammatory responses and contributing to joint inflammation and pain [41,42]. Elevated levels of low-molecular-weight (LMW) HA, resulting from the degradation of high-molecular-weight (HMW) HA, can act as damage-associated molecular patterns (DAMPs), further stimulating the immune response and potentially leading to arthritic manifestations [40,41]. Recently, Li et al. investigated the predictive value of HA levels in assessing disease severity in patients with SARS-CoV-2 infection during the post-COVID-19 era [43]. The study included 217 patients admitted to Beijing Ditan Hospital between July and October 2023. Their findings demonstrated that elevated serum HA levels were significantly associated with more severe clinical manifestations of SARS-CoV-2 infection, including heightened systemic inflammatory markers, extensive pulmonary involvement, prolonged disease duration, and increased risk of respiratory failure and mortality. Notably, the study also reported that HA levels did not fully normalize following clinical recovery, suggesting persistent dysregulation of HA metabolism beyond the acute phase of infection [43]. The inflammatory cytokines released during COVID-19 can promote the synthesis of HA, creating a feedback loop that exacerbates endothelial injury and joint inflammation [42,44]. Thus, the interplay between COVID-19-induced changes in HA levels and the inflammatory processes may play a crucial role in the development of arthritic symptoms in patients recovering from the virus.

## 5. Alterations in Bone and Collagen Biomarkers in Post-COVID-19

The COVID-19 pandemic has revealed significant impacts in bone and cartilage metabolism through distinct biochemical pathways. Accumulating evidence suggests that SARS-CoV-2 infection initiates systemic inflammatory responses and cytokine dysregulation, significantly influencing musculoskeletal health [45,46]. This disruption is measurable through various biomarkers involved in bone formation, mineralization, and cartilage remodeling. Notably, biomarkers such as serum procollagen type I N-propeptide (PINP) [47,48], serum total alkaline phosphatase (ALP) [17,49], osteocalcin [50,51], matrix metalloproteinases (MMP-3 and MMP-9) [52,53,54], and osteopontin (OPN) [55,56] have emerged as critical indicators reflecting the extent of COVID-19’s impact on skeletal and joint tissues. Investigating the roles and fluctuations of these biomarkers provides insights into the complex pathophysiology of musculoskeletal complications post-COVID-19 and highlights their potential as diagnostic and prognostic tools in managing long-term outcomes for affected individuals.

### 5.1. PINP (PIIINP)

Serum procollagen type I N-propeptide (PINP) is designated as a reference marker of bone and collagen formation. It is generated and released into the bloodstream when procollagen is converted to collagen. Procollagen molecules are generated by osteoblasts, in which there are pro-peptide extensions at the amino terminals. These terminals are cleaved off and released into the bloodstream when the collagen molecules start to form the osteoid matrix [47]. PINP is constructed of three subunits chains of type 1 procollagen, including two pro- α1 chains and a pro-α2 chain [57]. These are non-covalently linked and are produced in equal amounts when collagen is deposited in bone tissue, making them viable markers that reflect the rate of bone formation [47]. In Chavassieux’s work, we see that serum PINP was found to be significantly correlated to the histomorphometric measurements of bone formation [48] and, for this reason, has been designated a reference marker of bone formation in osteoporosis by the Internation Osteoporosis Foundation (IOF) and the International Federation of Clinical Chemistry and Laboratory Medicine (IFCC). PINP can be used as a marker to track the progression of multiple disease states, such as Paget’s disease of bone, or even the outcome of bisphosphonate therapy, to determine the efficacy of the treatment plan [47].

Recent studies have shown that serum biomarkers of collagen formation and tissue remodeling are elevated in COVID-19 patients, particularly those developing severe or interstitial lung disease (ILD). Procollagen type III N-terminal peptide (PIIINP) levels were significantly higher in patients with fatal outcomes and those who developed ILD [45,58]. Similarly, hyaluronic acid (HA) and type VI collagen formation were associated with disease severity and mortality [58,59]. These biomarkers, along with laminin and type IV collagen, were also linked to the progression of post-COVID-19 pulmonary fibrosis [60]. Additionally, markers of collagen crosslinking, fibrin formation, and neutrophil activity were elevated in COVID-19 patients [45]. These findings suggest that serum biomarkers of extracellular matrix remodeling may serve as valuable indicators of COVID-19 severity and potential complications; however, there is limited insight and work on how PINP levels in serum correlate with musculoskeletal health affected by COVID-19.

### 5.2. Serum Total Alkaline Phosphatase (ALP)

Serum total alkaline phosphatase (ALP) is a zinc metalloprotein enzyme that separates terminal phosphate groups from organic phosphate esters. It is a crucial enzyme involved in bone metabolism, particularly in the context of musculoskeletal health [61]. It comprises several isoenzymes, including bone-specific ALP, which is associated with osteoblastic activity. ALP levels can increase due to various factors, primarily obstructive liver disease and metabolic bone disorders [62,63]. It plays a vital role in bone mineralization by hydrolyzing inorganic pyrophosphate, a known inhibitor of mineral formation, thereby facilitating the accumulation of hydroxyapatite crystals within the extracellular matrix of bones [63]. Age and gender influence ALP levels, with postmenopausal women showing higher bone-specific ALP compared to premenopausal women [64]. ALP activity positively correlates with age in the general population, suggesting its potential as a biomarker of aging [65]. Elevated ALP levels can indicate various conditions, including Paget’s disease, rickets, osteomalacia, and certain cancers, making it a useful diagnostic tool in metabolic bone diseases [64,66]. Conversely, low levels of ALP may signal conditions like hypophosphatasia, characterized by impaired bone mineralization and associated musculoskeletal complications [61]. Thus, monitoring serum ALP levels serves as an important diagnostic tool in assessing bone health and managing musculoskeletal issues [66].

Recent studies have investigated the impact of COVID-19 on bone health and liver function. Research suggests that COVID-19 may affect bone remodeling, potentially leading to osteopenia or osteoporosis, as indicated by elevated bone alkaline phosphatase levels and reduced bone mineral density in post-COVID patients [49]. Alkaline phosphatase (ALP) has been proposed as a biomarker for osteoporosis diagnosis during the pandemic [67]. The infection can induce a pro-inflammatory state characterized by elevated levels of cytokines, which disrupts the balance of bone metabolism. Specifically, these inflammatory cytokines promote osteoclastogenesis, the process by which bone-resorbing cells (osteoclasts) are formed while simultaneously inhibiting the function of osteoblasts, the cells responsible for bone formation [68]. This imbalance can decrease bone mineral density (BMD) and increase the risk of fractures among COVID-19 patients [17]. Research indicates that patients recovering from COVID-19 often exhibit elevated ALP levels. For instance, a study found that post-COVID-19 patients had significantly higher serum ALP levels than non-COVID-19 individuals, indicating a potential link between the infection and bone remodeling processes [49]. Additionally, the relationship between ALP levels and BMD has been noted, with higher ALP levels correlating with lower BMD in some studies, further emphasizing the importance of monitoring these biomarkers in COVID-19 patients [17]. Additionally, a syndrome of cholangiopathy characterized by markedly elevated serum ALP has also been observed in patients recovering from severe COVID-19, potentially resulting in progressive biliary injury and liver failure [69]. However, high-dose vitamin D supplementation has shown promise in improving ALP markers among COVID-19 patients, suggesting a potential therapeutic approach [70]. These findings highlight the complex relationship between COVID-19, bone metabolism, and liver function, warranting further investigation into long-term consequences and treatment strategies.

### 5.3. Osteocalcin

Osteocalcin (OC) is a non-collagenous, vitamin K-dependent protein produced primarily by osteoblasts, playing a crucial role in bone metabolism and energy regulation. It contains three residues of γ-carboxyglutamic acid, which enable it to bind calcium and hydroxyapatite, thus influencing bone mineralization [71]. Traditionally, osteocalcin was thought to inhibit bone formation; however, a few studies suggest it also regulates osteoblast and osteoclast activity [71,72]. The uncarboxylated form of osteocalcin has been shown to function as a hormone, impacting glucose metabolism by promoting pancreatic β-cell proliferation and insulin secretion, as well as influencing adiponectin production in adipose tissue [71,73,74]. Research indicates that osteocalcin may also have extra-skeletal effects, including roles in cognition and male fertility [75]. While animal studies have demonstrated its hormonal functions, human studies have produced mixed results, often failing to differentiate between the total and uncarboxylated forms of osteocalcin or account for dietary vitamin K intake, which affects carboxylation levels [74]. Consequently, the precise role of osteocalcin in human glucose metabolism remains unclear, necessitating further investigation to establish its potential as a therapeutic target for metabolic disorders [76,77].

Osteocalcin levels are significantly affected in patients with COVID-19, particularly in those with moderate to severe disease. Studies have shown that serum osteocalcin levels are reduced in COVID-19 patients compared to healthy controls, indicating a negative impact on bone metabolism due to the infection [50]. This reduction in osteocalcin is associated with increased bone resorption and altered bone turnover, which may be exacerbated by the inflammatory response triggered by the virus [46]. Furthermore, a cytokine storm in severe COVID-19 cases contributes to the downregulation of osteocalcin, as inflammatory cytokines can enhance osteoclast activity while inhibiting osteoblast function [46]. A study focusing on non-severe COVID-19 patients found that osteocalcin levels were significantly lower, suggesting that even mild cases can disrupt normal bone metabolism [51]. Additionally, in critically ill COVID-19 patients, low circulating osteocalcin was identified as a good marker for stress hyperglycemia, which was associated with longer ICU stays and higher amounts of glucose delivered through artificial nutrition [78]. Overall, the evidence indicates that COVID-19 negatively influences osteocalcin levels, which may lead to increased bone fragility and a higher risk of fractures in affected individuals [46].

### 5.4. MMP-3 and MMP-9

Matrix metalloproteinases (MMPs) are a family of zinc-dependent enzymes that play critical roles in the degradation of extracellular matrix (ECM) components, particularly in osteoarthritis (OA) and rheumatoid arthritis (RA). Among the various MMPs, MMP-3 and MMP-9 have been extensively studied due to their significant involvement in the pathogenesis of these diseases. MMP-3, also known as stromelysin-1, is produced by synovial fibroblasts and chondrocytes and is capable of degrading a wide range of ECM components, including aggrecan and type IX collagen. It is highly expressed in normal and early OA cartilage but shows a marked decrease in late-stage disease [79,80]. This suggests that MMP-3 may initially contribute to cartilage remodeling but becomes less active as the disease progresses. Studies have indicated that MMP-3 is essential for generating specific aggrecan and collagen breakdown products, implicating it in the destruction of collagen through the activation of collagenase activity [79]. Furthermore, MMP-3 levels have been correlated with disease activity in RA, serving as a potential biomarker for joint damage [81]. Singh’s research highlights that serum MMP-3 levels are significantly elevated in patients with primary knee osteoarthritis compared to healthy controls, indicating its potential as a diagnostic marker for early OA [82].

MMP-9, also referred to as gelatinase B, is primarily involved in the degradation of type IV and type V collagen, as well as gelatin. It is not typically expressed in normal cartilage but is significantly upregulated in moderate to late stages of OA [79,83]. MMP-9 is closely associated with inflammatory cytokines such as interleukin-1 (IL-1) and IL-6, which promote its production in response to joint inflammation [84]. Elevated levels of MMP-9 in synovial fluid have been linked to increased angiogenesis and accelerated disease progression in RA. MMP-9 has been shown to facilitate dendritic cell migration, which is crucial for the immune response in RA, further linking its activity to the inflammatory processes that exacerbate joint damage [85]. The roles of MMP-3 and MMP-9 in arthritis are interconnected through their contributions to cartilage degradation and inflammation, making them pivotal in the pathophysiology of these diseases and potential targets for therapeutic intervention [79,80].

Matrix metalloproteinases (MMPs) have been identified as significant biomarkers in the context of COVID-19, with various studies highlighting their altered levels during infection and their implications for disease severity and outcomes. For instance, in a recent work, MMP-3 was shown to be significantly elevated in COVID-19 patients compared to healthy controls, indicating its potential role in diagnosing the disease [54]. Similarly, it has been reported that serum MMP-3 levels increased progressively with the severity of COVID-19, while MMP-9 levels were significantly higher in patients with severe forms of the disease, although they did not correlate directly with severity [53]. In D. Avila-Mesquita’s work, MMP-9 levels were found to be increased by 195.4% in COVID-19 patients compared to controls, and both MMP-2 and MMP-9 levels were associated with mortality risk [52]. Furthermore, it was identified that the MMP-9/BDNF ratio could be used as a potential predictor of severe COVID-19 outcomes, demonstrating its utility in differentiating between disease stages [86]. The involvement of MMPs in COVID-19 extends beyond mere biomarkers, as they are implicated in the pathophysiological mechanisms of the disease. It was highlighted that MMP-9 levels were significantly higher in COVID-19 patients with neurological symptoms, suggesting a link between MMP activity and neurological complications [87]. Additionally, there is evidence that increased circulating MMP-9 levels may persist even after clinical recovery from COVID-19, indicating their potential role in long-term complications associated with the disease [88]. D. Avila-Mesquita’s study emphasized that elevated MMP levels could serve as prognostic indicators of in-hospital mortality, suggesting that targeting these MMPs pharmacologically may offer therapeutic benefits [52]. Collectively, these findings underscore the critical role of MMPs in COVID-19, not only as biomarkers for disease severity but also as potential therapeutic targets to mitigate the disease’s impact.

### 5.5. Osteopontin

Osteopontin (OPN) is a multifunctional phosphorylated glycoprotein that plays a critical role in various biological processes, particularly in bone metabolism and musculoskeletal health. It is a member of the small integrin-binding ligand N-linked glycoprotein (SIBLING) family and is primarily secreted by osteoblasts, osteoclasts, and chondrocytes, contributing to cell adhesion, migration, and survival [89]. OPN facilitates the attachment of osteoclasts to the bone matrix through interactions with integrins such as αvβ3 and CD44, which are essential for bone resorption. In the context of osteoarthritis (OA), OPN levels are significantly elevated in both plasma and synovial fluid of patients, correlating positively with disease severity, indicating its potential as a biomarker for OA progression [90]. Furthermore, studies have shown that OPN is involved in the pathological processes of OA by promoting inflammation and cartilage degradation, as it is upregulated in osteoarthritic chondrocytes [91]. Lund’s work highlights OPN’s role in mediating cell migration, adhesion, and survival, emphasizing its involvement in chronic inflammatory diseases, including OA [92]. Additionally, it has been proposed that OPN could be considered a common denominator in the immunopathology of rheumatic diseases, including OA and rheumatoid arthritis (RA), due to its significant presence in inflammatory processes [93]. In osteopontin-deficient mice, there is a notable reduction in osteoclast activity and bone resorption, highlighting OPN’s role in maintaining bone homeostasis and its involvement in the development of musculoskeletal disorders [94]. Moreover, OPN’s phosphorylation status has been linked to the regulation of matrix metalloproteinase 13 (MMP-13), an enzyme that contributes to cartilage breakdown in OA [95]. Overall, OPN serves as a crucial mediator in the interplay between bone remodeling and inflammatory processes, making it a significant factor in musculoskeletal diseases such as OA and osteoporosis [96].

Osteopontin (OPN) has emerged as a significant biomarker in the context of COVID-19, particularly concerning disease severity and outcomes. Elevated levels of OPN have been consistently observed in patients with severe COVID-19, correlating with inflammatory markers that reflect the severity of the disease. For instance, a study by Hayek et al. reported that hospitalized COVID-19 patients had mean OPN levels of 96.63 ng/mL compared to 16.56 ng/mL in healthy controls, indicating a strong association with adverse outcomes [55]. Similarly, another study found that baseline plasma OPN levels exceeding 437 ng/mL predicted severe disease evolution with 53% sensitivity and 83% specificity [56]. Varım et al. also demonstrated that critically ill patients exhibited significantly higher OPN levels (13.75 ng/mL) compared to non-critically ill patients (9.85 ng/mL), suggesting that OPN may serve as a valuable marker for predicting COVID-19 severity [97]. In pediatric populations, it was highlighted that OPN levels were significantly elevated in children diagnosed with multisystem inflammatory syndrome (MIS-C) compared to those with mild or asymptomatic COVID-19, reinforcing its potential role as a biomarker for severe inflammatory responses in children [98]. Furthermore, it was noted that high plasma levels of OPN were associated with inflammatory markers in COVID-19 pneumonia, emphasizing its relevance in the inflammatory cascade [99]. Collectively, these findings underscore the potential of OPN as a prognostic tool in COVID-19, warranting further investigation into its mechanisms and clinical applications.

## 6. Alteration in Muscle Biomarkers Post-COVID-19

As mentioned above, COVID-19 can cause rapid muscle loss and acute sarcopenia, parallel to cartilage and bone loss. Clinical studies have documented marked reductions in muscle mass, strength, and function, with some patients losing 5–10% of body weight [20,100]. These deficits substantially increase the risk of long-term disability, fractures, and overall musculoskeletal degeneration. Beyond structural loss, recent evidence indicates that several muscle-related growth factors, myogenic proteins, and muscle enzymes, such as creatine kinase, are dysregulated during both the acute and post-acute stages of COVID-19. In particular, insulin-like growth factor 1 (IGF-1), myostatin, and follistatin have been documented, reflecting disrupted anabolic–catabolic signaling balance. Such molecular alterations may impair myogenesis, exacerbate proteolysis, and hinder muscle regeneration, thereby contributing to persistent muscle atrophy and functional decline in post-COVID-19 individuals.

### 6.1. Myostatin

Myostatin is a muscle-derived growth differentiation factor that negatively regulates skeletal muscle mass by inhibiting protein synthesis and promoting degradation. Produced mainly in skeletal muscle, it also influences metabolic pathways and contributes to oxidative stress [101]. Elevated myostatin is linked to muscle wasting, frailty, and conditions such as obesity, COPD, sepsis, and sarcopenia and is considered a potential biomarker for muscle impairment and rehabilitation needs [102,103]. Multiple post-COVID investigations report elevated myostatin levels after COVID-19 infection and link higher myostatin concentrations to worse outcomes relevant to muscle loss and prolonged recovery. Mińko et al. found a positive correlation between myostatin and both length of hospitalization and duration of rehabilitation in post-COVID patients [102]. At the same time, Visconti et al. identified myostatin as an independent factor associated with persistent post-COVID fatigue [104]. These findings support a pathway in which critical illness, immobilization, and catabolic inflammation during COVID-19 raise myostatin, promoting acute sarcopenia and prolonged functional impairment; accordingly, increased myostatin after COVID-19 is associated with longer rehabilitation, greater muscle wasting risk, and poorer functional recovery [102,103,104].

### 6.2. IGF-1

Insulin-like growth factor 1 (IGF-1) is a circulating peptide hormone structurally related to insulin that mediates many growth hormone effects, promoting cell proliferation, survival, protein synthesis, and tissue growth through activation of PI3K–AKT and MAPK signaling pathways [105,106]. Furthermore, it plays a key role in muscle development, repair, and maintenance by stimulating protein synthesis, activating satellite cells, and reducing protein breakdown [100]. Its levels decline with age and contribute to muscle atrophy [100].

Several COVID-19 studies report alterations in IGF-1 during infection with implications for muscle wasting. Zhao et al. found that IGF1 signaling from immune cells was altered during recovery from COVID-19 and that serum IGF1 declined after viral clearance [107], while Feizollahi et al. reported no overall difference in IGF-1 between severe COVID-19 patients and controls but observed correlations of IGF-1 with age and clinical features [105]. Ilias et al. observed lower age-normalized IGF-1 in critically ill and non-surviving COVID-19 patients and inverse correlations of IGF-1 with markers of severity and with age in their cohort, suggesting that low IGF-1 associates with worse outcomes [106]. Because IGF-1 is a key anabolic factor for muscle protein synthesis, reduced circulating or tissue IGF-1 in severe or protracted COVID-19 may contribute to sarcopenia and muscle loss [105,106].

### 6.3. Follistatin

Follistatin is a physiological inhibitor of activins (TGF-β superfamily ligands). It binds activins, promotes their endocytosis and proteolytic degradation, and thereby limits activin bioavailability and signaling; while expressed broadly in healthy tissues, its levels rise in immuno-inflammatory states as part of stress responses [107,108]. In acute COVID-19 pathophysiology, the activin/follistatin axis is perturbed, with follistatin acting as a key counter-regulator of heightened activin signaling [107,108].

Synolaki et al. found that serum follistatin rises markedly during days 7–28 from symptom onset. It shows the strongest individual prognostic performance among measured biomarkers and is incorporated at a threshold > 6235 pg/mL in the 10-point FACT-CLINYCoD mortality score. Elevations were independently associated with in-hospital death across initial and validation cohorts [107]. Divolis et al. similarly noted that elevated circulating follistatin in COVID-19 correlates with disease severity and with neutrophil inflammatory transcriptional programs, underscoring axis dysregulation [109]. These studies focus on the acute/hospital phase; they do not report post-COVID follistatin trajectories or evaluate sarcopenia outcomes, so within these data, a direct correlation between post-COVID follistatin levels and sarcopenia cannot be established [107,108,109].

### 6.4. Creatine Kinase

Creatine kinase (CK) is an intracellular enzyme primarily found in skeletal muscle, heart, and brain that catalyzes the reversible transfer of a high-energy phosphate group from phosphocreatine to ADP to regenerate ATP, thus sustaining short bursts of high-energy demand in muscle cells [110,111,112]. Elevations in serum CK reflect muscle membrane disruption or increased muscle cell permeability; therefore, it is used as a biomarker for muscle injury and myopathies [113]. CK isoforms and kinetics make CK a practical lab measure to track muscle damage and recovery in clinical practice [113].

Several studies have reported elevated creatine kinase (CK) levels during acute COVID-19 infection, correlating with adverse clinical outcomes and suggesting the occurrence of transient muscle injury or catabolic muscle responses. Orsucci et al. (2021) conducted a retrospective cohort study of 331 hospitalized patients during Italy’s first COVID-19 wave, stratifying cases into “severe” (*n* = 99) and “mild” (*n* = 232) outcome groups [111]. CK levels at admission were significantly higher in patients with severe outcomes (median 126 U/L; range 10–1672) compared with those with mild disease (median 82 U/L; range 12–1499; *p* = 0.01), and hyperCKemia (>200 U/L) was associated with poorer prognosis. Multivariate regression confirmed CK elevation as an independent predictor of disease severity. Notably, CK elevations were generally transient, resolving during hospitalization in most patients irrespective of outcome. Similarly, Friedman et al. (2022) performed a retrospective single-center cohort study of 289 hospitalized patients (mean age 68.5 years; 50.2% men; 90.7% African American) and found elevated CK (>220 U/L) in 45.7% of cases, with 18.0% reporting myalgia and 31.8% reporting subjective weakness [112]. Elevated CK was significantly associated with more severe disease, including the need for intubation, intensive care, and/or death. However, no association was observed between CK levels and skeletal muscle symptoms or other laboratory markers. In addition, Laches et al. (2023) reported an extreme case of COVID-19–associated rhabdomyolysis in a male patient in his early thirties, characterized by profound skeletal muscle breakdown and a peak CK level of 1,650,000 U/L, far exceeding typical values reported in viral-associated rhabdomyolysis [113]. Collectively, these findings underscore CK as a potential prognostic biomarker in COVID-19 and highlight the capacity of SARS-CoV-2 to induce substantial, and in rare cases extreme, muscle injury. It is proposed that virus-triggered systemic inflammation and cytokine-mediated catabolism likely drive transient hyperCKemia and can lead to sarcopenia if muscle wasting occurs during prolonged illness or recovery [111,112].

## 7. Discussion

The COVID-19 pandemic has revealed a multitude of complications beyond the respiratory symptoms traditionally associated with the virus. Among these, musculoskeletal (MSK) concerns have emerged as significant issues affecting a substantial number of patients. The dysregulation of various biomarkers in COVID-19 patients provides critical insights into the underlying mechanisms contributing to these MSK complications. Research indicates that individuals recovering from COVID-19 frequently report symptoms such as fatigue, muscle pain, and joint discomfort, which can persist long after the acute phase of the illness [14]. These symptoms are particularly pronounced in the elderly and those with pre-existing chronic conditions, who are already at a heightened risk for both COVID-19 and MSK disorders. The interplay between the inflammatory response triggered by the virus and the existing vulnerabilities in these populations may exacerbate the severity and duration of musculoskeletal issues.

Biomarkers such as cartilage oligomeric matrix protein (COMP), hyaluronic acid (HA), osteocalcin, and procollagen type I N-terminal peptide (PINP) have been identified as key indicators of musculoskeletal health and are notably affected in COVID-19 patients (Table 1). For instance, decreased serum levels of COMP have been observed in patients with severe COVID-19, suggesting a potential link to cartilage degradation and osteoarthritis progression [34]. This reduction in COMP may reflect the underlying cartilage damage that contributes to joint pain and dysfunction, highlighting the importance of monitoring this biomarker in the context of MSK health. Similarly, HA, which plays a crucial role in joint lubrication and cartilage health, has been shown to undergo significant alterations during COVID-19 infection. The dysregulation of HA metabolism, characterized by the accumulation of low-molecular-weight HA fragments, may contribute to joint inflammation and pain, further complicating the musculoskeletal landscape in affected individuals [39,40]. Elevated levels of inflammatory cytokines during COVID-19 can promote HA synthesis, creating a feedback loop that exacerbates both endothelial injury and joint inflammation.

Osteocalcin, a marker of bone metabolism, is also significantly impacted in COVID-19 patients. Studies have demonstrated that serum osteocalcin levels are reduced in those with moderate to severe disease, indicating a negative influence on bone turnover and potentially leading to increased bone fragility [50]. The inflammatory response associated with COVID-19 may enhance osteoclast activity while inhibiting osteoblast function, resulting in a detrimental imbalance in bone remodeling processes [46]. This disruption could explain the increased risk of fractures and other bone-related complications observed in post-COVID patients. Additionally, serum procollagen type I N-terminal peptide (PINP) has emerged as a significant biomarker in the context of COVID-19. PINP is a marker of collagen synthesis and is indicative of ongoing tissue remodeling. Elevated levels of PINP have been associated with severe COVID-19 outcomes, particularly in patients who develop interstitial lung disease [58]. The increase in PINP levels may reflect heightened collagen turnover and remodeling processes in response to the inflammatory state induced by the virus. This dysregulation can contribute to altered musculoskeletal health, as excessive collagen degradation or impaired synthesis can lead to joint instability and pain. Furthermore, serum total alkaline phosphatase (ALP) is a crucial enzyme involved in bone metabolism and has been shown to be elevated in COVID-19 patients. Research suggests that COVID-19 may affect bone remodeling, potentially leading to osteopenia or osteoporosis, as indicated by elevated ALP levels and reduced bone mineral density in post-COVID patients [49]. The infection can induce a pro-inflammatory state characterized by elevated levels of cytokines, which disrupt the balance of bone metabolism. Specifically, these inflammatory cytokines promote osteoclastogenesis while inhibiting osteoblast function, resulting in decreased bone mineral density and an increased risk of fractures among COVID-19 patients [17]. Monitoring ALP levels in COVID-19 patients is essential, as elevated ALP may indicate ongoing bone remodeling processes that could predispose individuals to musculoskeletal complications. Osteopontin (OPN) has also emerged as a significant biomarker in the context of COVID-19, particularly concerning disease severity and outcomes. Elevated levels of OPN have been consistently observed in patients with severe COVID-19, correlating with inflammatory markers that reflect the severity of the disease [55]. For instance, hospitalized COVID-19 patients exhibited mean OPN levels significantly higher than those in healthy controls, indicating a strong association with adverse outcomes [55]. In the context of musculoskeletal health, OPN is involved in the pathological processes of osteoarthritis (OA) by promoting inflammation and cartilage degradation. Its presence in the synovial fluid and plasma of OA patients correlates positively with disease severity, suggesting that OPN may serve as a biomarker for OA progression [90]. The inflammatory response triggered by COVID-19 may exacerbate the effects of OPN, leading to increased joint inflammation and pain, thereby contributing to the musculoskeletal complications observed in post-COVID patients. Similarly, as with cartilage and bone, muscle loss is also affected in COVID-19 patients. Prolonged hospitalization, systemic inflammation, and reduced physical activity during the acute phase of illness contribute to rapid skeletal muscle atrophy. Myostatin, a negative regulator of muscle growth, has been reported to be upregulated in severe cases, exacerbating protein breakdown and limiting hypertrophy [102,103]. In contrast, follistatin, a myostatin antagonist, may be reduced, diminishing its protective effect against muscle wasting [107,108]. Elevated serum creatine kinase (CK) levels in COVID-19 patients indicate ongoing muscle damage and are often associated with severe myopathy [111,112]. This muscle wasting not only delays functional recovery but also worsens overall prognosis, as reduced muscle reserves are linked to increased morbidity and mortality.

At present, no longitudinal cohort studies have specifically examined the progression of musculoskeletal (MSK) symptoms in COVID-19 patients. Furthermore, there are no established clinical diagnostic parameters currently in use to definitively identify osteoarthritis (OA), bone loss, or sarcopenia in this population. The limited available studies have primarily relied on subjective measures, such as pain scales, to assess disease burden, while objective diagnostic criteria remain absent [114]. Recent studies focused on indirect biomarkers; however, these lack the specificity and sensitivity required for definitive diagnosis. Future studies should analyze the genomic and transcriptomic profiles of muscle tissue, using approaches similar to those applied in other disease models [115,116,117], to better elucidate the molecular mechanisms underlying COVID-19–related MSK complications. This gap underscores the urgent need for standardized, validated diagnostic tools and longitudinal monitoring to characterize and manage post-COVID musculoskeletal complications accurately.

## 8. Potential Translational Implications of Summarize Biomarkers in COVID-19 Infection

The findings summarized above have important translational implications for the management of COVID-19-associated MSK complications. The identification of altered biomarkers such as osteocalcin, PINP, ALP, OPN, COMP, HA, IGF-1, myostatin, follistatin, and creatine kinase can provide a foundation for developing minimally invasive diagnostic tools to detect early bone, cartilage, and muscle involvement in COVID-19 patients. These biomarkers could be integrated into clinical screening protocols to identify individuals at higher risk for long-term MSK impairment, enabling early intervention. Furthermore, these insights support the development of targeted rehabilitation strategies that combine physical therapy, nutritional supplementation, and pharmacologic modulation to promote MSK recovery. Finally, the integration of biomarker monitoring into longitudinal follow-up programs could guide personalized treatment approaches, reduce the risk of disability, and improve quality of life in COVID-19 survivors.

## 9. Impact of COVID-19 Vaccination on MSK

Across randomized trials and meta-analyses, COVID-19 vaccines significantly reduce incident COVID-19 and are generally well tolerated. Pooled analyses show strong protection overall, with mRNA vaccines most efficacious, followed by inactivated and non-replicating viral vector platforms (mRNA ≈ 92%, inactivated ≈ 81%, vector ≈ 67%) [118]. Vaccination also lowers the risk of developing long COVID symptoms at the population level, with calibrated sub-distribution hazard ratios ranging roughly 0.48–0.71 across large UK/Spain/Estonia datasets [119]. The most common adverse effects are transient local and systemic reactions, such as injection-site pain, swelling, erythema, fatigue, headache, fever, myalgia, and arthralgia. While serious adverse events are uncommon, a single trial report noted paroxysmal ventricular arrhythmia as a serious event [118]. Reactogenicity is typically higher after dose 2 and in younger adults for mRNA vaccines [118].

Long-term musculoskeletal outcomes specific to vaccination remain less clearly characterized in pivotal trials and early real-world analyses; however, available evidence consistently supports a favorable safety profile with very rare unexpected serious events and mainly mild local/systemic effects [118,119]. Theoretical longer-term considerations include monitoring for inflammation or autoimmunity with mRNA platforms and local cytokine/adjuvant effects with inactivated or subunit vaccines. To data, clinical evidence does not indicate a significant risk to MSK harm [118]. Notably, by lowering both acute COVID-19 and the risk of long COVID, which itself features a musculoskeletal symptom cluster (e.g., myalgia and joint pain), vaccinations may indirectly reduce persistent MSK symptom burden at the population level [119,120]. However, dedicated studies employing standardized musculoskeletal assessment tools and longitudinal follow-up are needed to directly evaluate post-vaccination MSK outcomes and differentiate them from those attributable to SARS-CoV-2 infection.

## 10. Conclusions

**Figure 1:**
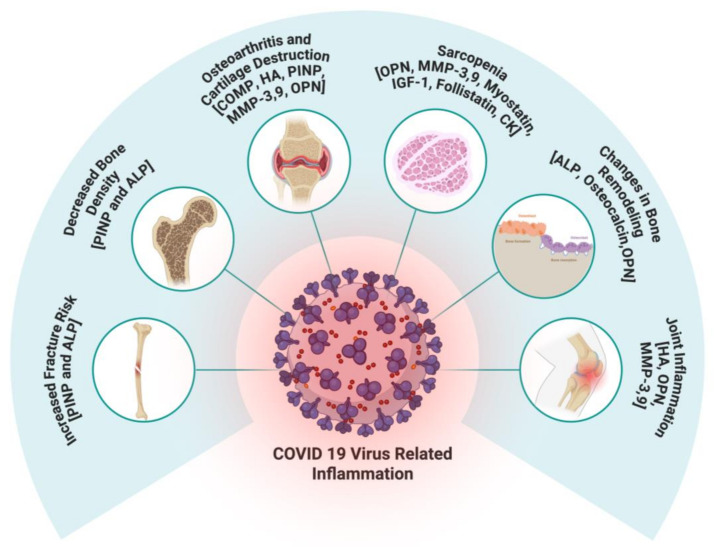
Schematic illustration showing the impact of COVID-19-related inflammation on musculoskeletal health through alterations in key circulating biomarkers.

The musculoskeletal complications observed in COVID-19 patients are multifaceted and likely result from several factors, such as direct viral effects, inflammatory responses, and pre-existing vulnerabilities. The dysregulation of biomarkers such as COMP, HA, osteocalcin, PINP, ALP, and OPN provides valuable insights into the mechanisms underlying these complications (Figure 1). Similarly, muscle-related biomarkers, including IGF-1, myostatin, follistatin, and creatine kinase further highlight the profound impact of COVID-19 on skeletal muscle mass, function, and recovery potential. Future studies should focus on longitudinal studies to better understand the long-term implications of these biomarker changes on musculoskeletal health in COVID-19 survivors and potential therapeutic interventions to mitigate these effects. Addressing these concerns is essential for improving the quality of life for individuals recovering from COVID-19 and informing clinical practices in managing post-viral musculoskeletal disorders. Most importantly, there is a need for follow-up studies to understand the impact of COVID-19 vaccination on MSK.

## Figures and Tables

**Table 1:** Impact of COVID-19 on key biomarkers of musculoskeletal health.

Biomarker	Role in MSK Health	Alteration in COVID-19	Implications for MSK Health	References
Cartilage Oligomeric Matrix Protein (COMP)	Stabilizes collagen fiber network in articular cartilage; marker of cartilage degradation and osteoarthritis progression	Decreased serum COMP levels, especially in severe COVID-19 cases	Indicates cartilage degradation, increased osteoarthritis progression and joint pain	[26,33,34,35]
Hyaluronic Acid (HA)	Maintains synovial fluid viscosity and joint lubrication.	Dysregulated metabolism with accumulation of low-molecular-weight HA fragments	Joint inflammation and pain; exacerbates endothelial dysfunction and inflammatory responses	[26,39,40,41,42,43,44]
Procollagen Type I N-terminal Peptide (PINP)	Marker of bone and collagen formation; reflects bone formation rate	Elevated levels in severe COVID-19, especially with interstitial lung disease	Indicates excessive tissue remodeling; altered musculoskeletal remodeling	[45,47,48,58,59,60]
Serum Total Alkaline Phosphatase (ALP)	Enzyme involved in bone mineralization and remodeling	Elevated levels in COVID-19 patients	Associated with accelerated bone remodeling, decreased bone mineral density, increased fracture risk	[17,49,61,62,63,64,65,66,67,68,69,70]
Osteocalcin	Vitamin K-dependent protein regulating bone mineralization and turnover	Reduced serum levels in moderate to severe COVID-19 patients	Disrupted bone turnover, increased bone fragility, higher fracture risk	[46,50,51,78]
Matrix Metalloproteinase-3 (MMP-3)	Degrades extracellular matrix components; involved in cartilage remodeling and inflammation	Significantly elevated in COVID-19; levels increase with disease severity	Contributes to cartilage degradation; potential diagnostic marker for disease severity	[52,53,54,79,80,81,82]
Matrix Metalloproteinase-9 (MMP-9)	Degrades collagen types IV and V; linked to inflammation and immune response	Elevated in severe COVID-19; associated with inflammatory responses and mortality risk	Linked to cartilage degradation and inflammation; potential prognostic marker	[52,84,85,86,87,88]
Osteopontin (OPN)	Glycoprotein involved in bone resorption, inflammation, and cartilage degradation	Markedly increased in severe COVID-19 patients	Correlates with inflammation, joint damage, and musculoskeletal complications	[55,56,89,90,91,92,93,94,95,96,97,98,99]
Myostatin	Muscle-derived factor that inhibits protein synthesis and promotes breakdown; linked to wasting, frailty, and sarcopenia.	Elevated after COVID-19; associated with longer hospital stay, prolonged rehab, and fatigue.	Higher risk of muscle loss, longer recovery, and reduced function.	[101,102,104]
IGF-1	Hormone that promotes cell growth, protein synthesis, and anabolic processes; supports muscle, lung, liver, and brain health.	Reduced levels after COVID-19; lower in severe cases and non-survivors; inversely linked to age and severity.	Low IGF-1 may contribute to muscle loss and sarcopenia in severe/prolonged cases.	[100,105,106,107]
Follistatin	Inhibits activin signaling; rises during inflammation as a protective response.	Markedly elevated in acute COVID-19; linked to severity and higher mortality risk.	No direct post-COVID link to sarcopenia, but indicates severe systemic inflammation.	[107,108,109]
Creatine Kinase (CK)	Enzyme for ATP regeneration in muscle; marker of muscle injury.	Elevated in acute COVID-19; higher in severe cases; extreme rises in rhabdomyolysis.	Suggests muscle injury; persistent elevation may lead to loss of muscle mass and function.	[111,112,113]
